# Monitoring of cases of anosmia may help control the COVID-19 pandemic

**DOI:** 10.1016/j.bjorl.2021.02.001

**Published:** 2021-02-08

**Authors:** Marcel Menon Miyake, Wilma Terezinha Anselmo-Lima

**Affiliations:** a Hospital da Santa Casa de Misericórdia de São Paulo, Departamento de Otorrinolaringologia, São Paulo, SP, Brazil; b Universidade de São Paulo (USP), Faculdade de Medicina de Ribeirão Preto (FMRP), Ribeirão Preto, SP, Brazil

One of the most significant difficulties that governments worldwide are facing during the COVID-19 pandemic is the lack of accurate and rapid data on infected patients' evolution. Especially in Brazil, limited access to the real time - polymerase chain reaction (RT-PCR) test for most of the population and the difficulty of storage and processing samples result in underreporting and delay in the official statistics cases. The lack of information of an updated data regarding the evolution of the pandemic makes political decision-making immensely difficult. Accurate screening of patients would allow predicting the pandemic's growth with some confidence and contribute to the proper planning of health units in an imminent escalation in hospitalizations. Adequate planning is essential for establishing clear criteria for the intensification or relaxation of restrictive circulation measures, as largely discussed and criticized in the last year.

The monitoring of patients with sudden changes in smell and taste could be an essential tool to complement and maximize the surveillance system of new cases of COVID-19. The onset of the COVID-19 pandemic alerted for dysosmia associated or not with dysgeusia as a frequent symptom in patients with COVID-19, with an estimated prevalence of 62%.^1^ Besides, these sensory changes occur, in general, before and are more specific than other symptoms, such as dry cough and fever. Sudden changes in smell and taste may also be associated with different causes, but these symptoms in the current context are predictable to the Sars-Cov-2 virus. Sudden smell loss has 65% and 97% of sensitivity and specificity, respectively, in the diagnosis of COVID-19.^2^ These rates are comparable to PCR-RT rates (87% and 97%).^3^ Thus, it represents an advantage when considering an expenditure of resources and limited availability of PCR-RT for Sars-Cov-2. Besides, patients should be tested in a limited interval of time.

Based on this information, an article published in October 2020 in the Nature Communications^4^ by Pierron et al.*, suggests* that sudden olfactory changes could represent an early predictive marker of the evolution of COVID-19 cases. The study conducted in France, Italy and the United Kingdom with more than 6500 patients, observed a strong positive correlation between the number of patients with sudden olfactory and taste changes and the number of hospitalized cases, intensive care unit admission, and mortality associated with COVID-19. Also, the number of new cases of sudden dysosmia was an indicator capable of predicting the response to restrictive measures earlier, showing a peak in the number of new cases 4 days after the installation of the lockdown, than the currently used indexes, such as the number of emergency consultations that reached the apex after 11 days of lockdown, and the number of hospitalizations in emergency units, peaked only after 14 days. When comparing the evolution of olfactory changes of France and Italy, and the United Kingdom, the first two countries with more restrictive decrees reached an abrupt drop in the report of new cases of sudden dysosmia after the lockdown compared to the third, which presented a more gradual reduction of cases presumably due to the less severe restrictive policy.

Thus, the number of new cases of sudden dysosmia and dysgeusia represents a marker with high specificity, minimally invasive, and low cost in diagnosing COVID-19, besides presenting high capillarity capacity in a large portion of the population in a short period. The variation in the number of new cases of olfactory changes over the days may also be an indicator of fundamental importance for estimating the evolution of the pandemic in the coming days and weeks. In addition, it is able to early detect the impact of restrictive measures of circulation and specific events, such as meetings on festive dates, regarding the number of cases of COVID-19. Even with the beginning of the vaccination in our population, the effective control of COVID-19 will not be immediate, and it is essential that, during this period, we continue to evolve, applying new strategies to minimize the impact of this disease on our community. The adoption of a unified internet self-reported notification system of new cases of sudden dysosmia or dysgeusia, along with the contribution of otorhinolaryngologists and frontline physicians, would create a robust and low-cost database, that would help screening and fighting this pandemic. This system with accurate and up-to-date information would allow a more effective action of health authorities in a potential increase of new cases, minimizing the risks of overcrowding health units. Besides, the strategic planning of the intensification and relaxation of restrictive measures of circulation would be more efficient, being possible not only to control the escalation in the number of cases and hospitalizations, but also to avoid too strict and prolonged trade closure and closing of other activities that largely impacted the socio-economic activities in 2020.

## Conflicts of interest

The authors declare no conflicts of interest.
